# Editorial: Dysfunctional motivation in eating behaviors: a complex gene x environment interplay

**DOI:** 10.3389/fnbeh.2024.1367256

**Published:** 2024-02-09

**Authors:** Kirrilly Pursey, Lucy Babicola, Rossella Ventura, Matteo Di Segni

**Affiliations:** ^1^School of Health Sciences, College of Health, Medicine and Wellbeing, University of Newcastle, Callaghan, NSW, Australia; ^2^Food and Nutrition Research Program, Hunter Medical Research Institute, New Lambton Heights, NSW, Australia; ^3^Santa Lucia Foundation (IRCCS), Rome, Italy; ^4^Department of Psychology, Sapienza University of Rome, Rome, Italy; ^5^IRCCS San Raffaele, Rome, Italy

**Keywords:** eating disorder, food addiction, animal models, motivation, clinical studies

Feeding and eating behaviors are necessary for survival, requiring a balanced interaction between homeostatic needs and hedonic motivation. In some cases, these behaviors can become maladaptive, manifesting in potentially severe physical and psychological complications, including well-documented diagnosable eating and feeding disorders (FED) according to the Diagnostic and Statistical Manual of Mental Disorders (DSM-5). Feeding and eating disorders are becoming increasingly common worldwide, leading to considerable health and economic burden, including increased risk of mortality, reduced quality of life, and high healthcare costs. The concerning health and social impacts of FED highlights the significance of better understanding the underlying mechanisms of FED to support the development of more appropriate diagnostic tools and treatments. The etiology of FED is complex, involving neurobiological, genetic, social, and environmental factors. Cutting-edge pre-clinical investigations are currently used to understand the role of these factors, their interactions, and consequences in these disorders. This Research Topic Issue, consisting of 3 original research articles and 2 review articles, provides different “food for thought” with a journey between clinical matters and preclinical highlights in FED. This Topic covers complementary aspects, with the view to contributing to a deeper understanding of the complex “gene x environment interplay” in FED.

Vasilu's review focus on a less-recognized period for the development, escalation in severity, or relapse of disordered eating behaviors, the perinatal period. Maternal perinatal eating disorders, also termed “pregorexia,” pose significant medical and nutritional health risk for both the mother and baby. This review on an interesting nosological entity highlights the complexity in identifying, diagnosing, and treating people with an eating disorder in the perinatal period. The author concludes that raising awareness, early screening and detection with validated clinical instruments is essential for disordered eating during this high-risk period. Moreover, appropriate treatment is fundamental to improve the health of both the mother and baby. The author's review evidences important topics to be addressed by forthcoming research on this eating pathology.

The review by Passeri et al. explores the neurobiological and behavioral overlaps between drug addiction and compulsive food intake, commonly termed “food addiction.” Food addiction is not formally recognized in the DSM-5, however, its clinical features seem to fit with the diagnostic criteria for substance use disorders (i.e., high palatable, ultra-processed food may promote excessive consumption, usually associated with loss of control and craving). However, there is still significant debate as to whether the construct may more closely resemble a behavioral disorder (i.e., “uncontrolled eating” irrespective of nutritional composition). In this article, the authors highlight similarities and differences between food and drug addiction, investigating common neurobiological substrates within the mesoaccumbens dopamine system, and focusing on models of food addiction in rodents. The authors conclude that deepening the discussion on the concept of food addiction is of paramount importance to progress the field and for recognition as a diagnosable disorder.

Banu et al. highlight a mechanism by which serotonergic (5-HT) neural transmission—a critical neurotransmitter for feeding patterns and as pharmacological target for human therapies—modulates food motivation by capitalizing on an innovative *Drosophila* model. In particular, after reporting that prolonged food-deprivation increases food interaction, ingestion and sip duration in flies, authors evidence that optogenetic activation of serotonergic cells may suppress feeding regardless of physiological needs (i.e., fed and food-deprived), altering the microstructure of feeding behavior in absence of alteration in peripheral gustatory sensory responsiveness. Using intersection genetics, the study provides novel insight about how feeding patterns are influenced by *5-HT activity in specific CNS structures* in a hunger state-dependent manner. Moreover, evidencing how fine tuning of appetitive and consummatory behaviors are possibly mediated by opposite influences of different 5-HT (i.e., 5-HT1B and 5-HT7) receptors, the present work offers valuable preclinical insights for the investigation of specific mechanisms as possible targets of pharmacological therapies.

Lai and Chang's work uses a similar method to examine whether pharmacological modulation of animals' motivation toward food rewards and their associated cues is possible. Their hypothesis is centered on amygdala nuclei, a brain area critically involved in reward processing and particularly in encoding and retrieval of stimulus-reinforcement association. Thus, using a behavioral paradigm borrowed from addiction studies, authors show that activation through NMDA infusion of the amygdala nuclei (either BLA or CeA) abolishes cue-induced food-seeking after extinction in the absence of alteration in locomotor activity and feeding behavior when freely available. Interestingly, despite the amygdala mediating negative emotions processing, experimental exposure to acute stress before the reinstatement testing did not affect cue-induced motivation toward food rewards independently from anxiety and anhedonic-like states. Moreover, similar c-Fos activation in the amygdala and in key structures of the reward system (VTA, NAc) indicates that multiple systems may be engaged, depending on specific condition, in inducing pathological eating. Results reported in this work offer insight on the role of these brain structures in driving external cues and environmental stimuli to the hypothalamic feeding centers, in processing high-level sensory input and stimulus- value associations in dysfunctional eating.

Cue-induced motivation and goal-directed behavior toward food is also addressed in the article by Sood and Richard. In a preclinical (rat) model, the authors investigate the influence of sex on pathological eating behaviors associated with dysfunctional attribution of the expected value of rewards and associated cues, in driving food-directed maladaptive behaviors. Authors evidence how reward-seeking behaviors after devaluation by sensory-specific satiety (i.e., in absence of physiological needs) is more dampened in male subjects, suggesting that female rats are more prone to display devaluation-resistant or habit-like behaviors. In particular, sex differences reported were not due to differences in free access consumption, in sucrose devaluation itself, or post-test devaluation confirmation sessions. The model outlined in this article may be useful to investigate the neural and behavioral correlates of expected outcome value encoding and their influence on goal- directed behavior according to gender, resulting in a useful tool to investigate gender differences in susceptibility to eating disorders observed in clinical populations.

A common thread emerging from the papers included in this Research Topic across clinical matters (pregorexia and food addiction) and preclinical studies (murine and Drosophila models), highlights elements of differential susceptibility, as well as critical involvement of specific brain substrates such as neurotransmitters and structures, in FED that may manifest in specific diagnostic features. These collection of studies highlight how dysfunctional motivation in eating behaviors represent a continuously updating field that can benefit from the evaluation of new diagnostic criteria, as well as innovative pre-clinical models that can investigate the complex etiopathogenesis of these disorders. In addition, a better knowledge of the causal links between environmental and genetic factors in favoring the development of pathological phenotypes toward food could lead to a deeper understanding of the epigenetic mechanisms underlying these conditions. Further studies should be performed to identify peripheral biomarkers useful for clinical diagnosis, evaluation of the course of the pathology, and development of personalized treatments for FED.

## Author contributions

KP: Writing—original draft. LB: Writing—original draft. RV: Writing—review & editing. MD: Writing—review & editing.

